# Retraction Note: HGF and TGFβ1 differently influenced Wwox regulatory function on Twist program for mesenchymal-epithelial transition in bone metastatic versus parental breast carcinoma cells

**DOI:** 10.1186/s12943-020-01242-1

**Published:** 2020-08-15

**Authors:** Paola Bendinelli, Paola Maroni, Emanuela Matteucci, Maria Alfonsina Desiderio

**Affiliations:** 1 Dipartimento di Scienze Biomediche per la Salute, Molecular Pathology Laboratory, Università degli Studi di Milano, Milano, Italy; 2 Istituto Ortopedico Galeazzi, IRCCS, Milano, Italy

**Retraction note to: Mol Cancer 14, 112 (2015)**

**https://doi.org/10.1186/s12943-015-0389-y**

 The Editor-in-Chief has retracted this article [1] following an investigation by the Università degli Studi di Milano. The investigation found that both Figs. 2 and 3 have key problems of different types of manipulations of the vinculin WB used as loading control. In Fig. 2, the vinculin protein bands have been used in other publications from the same groups [2–4], while band rotation seems to have been performed in Fig. 3 to clone vinculin bands. The results of this study are thus unreliable.

The authors do not agree to this retraction.
